# Gender disparities in leadership of methodological research in health science

**DOI:** 10.1371/journal.pone.0359403

**Published:** 2026-09-29

**Authors:** Nicolette Christodoulakis, Daeria O. Lawson, Jessyca Silva, Jack Young, Laura N. Anderson, Harriette G. C. Van Spall, Carmen H. Logie, Lawrence Mbuagbaw

**Affiliations:** 1 Department of Health Research Methods, Evidence, and Impact, McMaster University, Hamilton, Ontario, Canada; 2 McMaster University Libraries, McMaster University, Hamilton, Ontario, Canada; 3 Department of Medicine, Faculty of Health Sciences, McMaster University, Hamilton, Ontario, Canada; 4 Factor Inwentash Faculty of Social Work, University of Toronto, Toronto, Ontario, Canada; 5 Women’s College Research Institute, Women’s College Hospital, Toronto, Ontario, Canada; 6 United Nations University Institute for Water Environment, and Health (UNU-INWEH), Richmond Hill, Ontario, Canada; 7 Biostatistics Unit, Father Sean O’Sullivan Research Centre, St Joseph’s Healthcare Hamilton, Hamilton, Ontario, Canada; 8 Department of Anesthesia, McMaster University, Hamilton, Ontario, Canada; 9 Department of Pediatrics, McMaster University, Hamilton, Ontario, Canada; 10 Centre for Development of Best Practices in Health (CDBPH), Yaoundé Central Hospital, Yaoundé, Cameroon; 11 Division of Epidemiology and Biostatistics, Department of Global Health, Stellenbosch University, Cape Town, South Africa; Instituto Tecnologico Autonomo de Mexico, MEXICO

## Abstract

**Objectives:**

Gender disparities in methodological studies are largely unexplored. We aimed to examine gender representation between first and last authorship positions in methodological research in health science.

**Methods:**

We searched for methodological articles as part of a large study focused on creating a methodological study reporting checklist. We extracted the names of the first and last authors of all included studies and determined their gender using Genderize.io, classifying authors as women if the gender probability was ≥ 60%. Our primary outcome was the proportion of studies with women in leadership roles (first or last author as separate outcomes). Secondary outcomes included the proportion of studies with both or either the first or last authors as women. We examined associations between women’s leadership and country income level, World Health Organization region, number of authors on the study team, and publication year.

**Results:**

Of the 1,375 included studies, the genders of 1,332 first authors and 1,334 last authors were determined. Among first authors, 675 (50.7%) were women, and 657 (49.3%) were men, indicating no gender difference. Among last authors, 486 (36.4%) were women, and 848 (63.6%) were men, with a statistically significant difference (χ² = 98.23, p < 0.001). Studies with women last authors had fewer listed co-authors than those with men (t = −3.63, p < 0.001; risk difference = −27.14%, 95% CI −32.50% to −21.77%). In adjusted models, women’s last authorship was negatively associated with number of authors (adjusted odds ratio [aOR]=0.93, 95% CI 0.90–0.97). Publication year was associated with both women first (aOR=1.03, 95% CI 1.01–1.05) and last (aOR=1.05, 95% CI 1.02–1.07) authorship.

**Conclusions:**

Our findings highlight a gender disparity in last authorship positions, with men more often in these roles. The association between women last authorship position and smaller author teams may reflect differences in collaborative structure and access to leadership opportunities in larger-scale methodological research. Addressing these disparities can help foster more inclusive research environments, especially within methodological research.

## Introduction

Methodological studies focus on examining or evaluating how research is designed, conducted, analyzed or reported, whether in published or unpublished work [1]. These studies aim to enhance the quality and transparency of research while reducing inefficiencies and research waste [2]. They address various aspects of the research process, including how questions are formulated, how findings are reported, and how analyses are conducted and interpreted [3–6]. In recent years, there has been an increase in the number of methodological studies conducted [1]. Despite their significance and the growing emphasis on equity in academic authorship, there remains a gap in understanding whether gender disparities exist within the leadership of methodological research. Addressing this gap is crucial not only for promoting inclusivity, but also strengthening research integrity, as diverse leadership can enhance the use of team members’ expertise, broaden the range of research questions and perspectives considered, and support more effective problem solving [7].

Sex and gender disparities in science have been recognized, with women historically underrepresented across various scientific fields, encountering barriers to career advancement, including both explicit and implicit biases [8,9]. These disparities are evident across various facets of academia and science including representation, compensation, grant success, authorship, awards, availability of funding opportunities, and rates of publication [9–12]. At the same time, there has been an increase in the number of women pursuing Science, Technology, Engineering, Mathematics, and Medicine (STEMM) subjects at the university level. A longitudinal study conducted between 1996 and 2018 examining the publication records of millions of researchers globally revealed an increasing proportion of women publishing, rising from 33% in 2000 to 40% in recent years [13]. However, despite this rise, disparities persist in career progression and publication output. The study also found that on average, men produced between 15% and 20% more publications than women [13]. The gaps are even more striking in historically male-dominated fields such as cardiovascular medicine, where women represent 15.6% of first authors, 12.9% of senior authors, and 11.4% of corresponding authors of trials [14]. High impact articles with women as primary/senior authors have half the citations than articles with men as lead/senior authors [15]. Further, within biomedical fields, men appeared to hold more senior positions as they were more likely to be the corresponding author of publications than women [13]. This trend is mirrored in findings from other studies which show that women continue to represent a minority among senior staff, facing obstacles such as limited representation in elite research groups and steering committees [16], slower promotion rates, and higher attrition rates from STEMM careers [17–20]. However, a recent cross-sectional study that examined data from ClinicalTrials.gov found that the proportion of women principal investigators significantly increased over time, rising from 27.0% in 2005 to 39.6% in 2023, highlighting progress alongside persisting disparities [21].

While gender disparities in various scientific disciplines have been examined, to our knowledge, an investigation into leadership within methodological research of health science remains unexplored. This study aims to address this gap by exploring gender differences in representation among leaders of methodological research.

## Materials and methods

### Study design

We conducted a secondary analysis of a methodologic study, basing our work off the search strategy developed for a review aimed at developing the MethodologIcal STudy reportIng Checklist (MISTIC) that has been previously described [22]. As indicators of research leadership, we assessed gender representation among first and last authors, which were operationalized as proxy measures of leadership. Although authorship practices may vary across research teams and collaborative settings, first and last authorship positions are commonly used in bibliometric research to examine leadership patterns [23].

### Search strategy

Searches were conducted across various databases including Cumulative Index to Nursing and Allied Health Literature, Cochrane Library, Excerpta Medica (Embase), MEDLINE and Web of Science. There were no restrictions placed on publication year, type, or language. Additionally, we searched for authors renowned for publishing methodological research, reviewed reference lists of relevant studies, explored methodological study repositories (Studies Within a Trial and Studies Within a Review), accessed preprints (bioRxiv and medRxiv), set up Google Alerts for relevant keywords (e.g., meta-epidemiology, research-on-research) and engaged with experts via email, meetings, and social media platforms such as ResearchGate and Twitter. These methods were informed by published literature and established practices [24,25]. The final search strategy was reviewed by two health sciences librarians at the Health Sciences Library at McMaster University, adhering to the Peer Review of Electronic Search Strategies framework [26]. The final search strategy sample for MEDLINE is detailed in S1 Table. This review is ongoing [27] and the search for the included studies in our analysis was updated on June 15, 2020.

### Eligible studies

All studies examining methods pertaining to the design, conduct, analysis, or reporting of human health research were considered eligible. Eligible records could be an abstract, protocol or a published methodological study. Only published protocols and final reports were included, while simulation studies, studies testing new statistical methods without a specific unit of analysis, and experimental studies of methods were excluded.

### Screening

Titles and abstracts of all articles identified in the search were screened in duplicate using Rayyan [28]. Full-text screening of eligible articles was performed using DistillerSR [29]. Any discrepancies between reviewers were resolved through discussion or by a third reviewer.

### Data extraction

Data extraction was performed using DistillerSR for the included studies and then exported for cleaning. Using the studies’ digital object identifiers (DOIs), we queried the bibliographic database Dimensions Analytics [30] and extracted the first name, last name, country of current organization/listed affiliation (where available), and author position (first or last) for each of the studies’ authors [31]. If an author’s name was missing or recorded only as initials after using Dimensions Analytics, we consulted full texts, Google Scholar profiles, ResearchGate profiles, ORCID profiles, Scopus profiles, and corresponding author emails to retrieve missing information. For group authorship, we recorded the last listed individual author as the last author instead of the group name. If the country of current affiliation was missing, we used the country listed in the published article. When multiple affiliations were listed, we recorded the first affiliation. For single-authored papers, we included the author in both the first and last author analyses. With the corresponding authors’ country of affiliation, we determined income levels based on the World Bank income classification [32] and geographical regions as per the World Health Organization (WHO). The number of authors on each paper was also computed.

For this analysis, the gender of both first and last authors were considered binary, categorized as either woman or man. We acknowledge this as a methodological constraint and as a limitation, as it excludes the broader range of gender identities that may be present among academic authors. Gender prediction was conducted using the validated software Genderize.io [33]. This software provides a predicted gender along with a probability for each name entered [33]. It allows for first names to be parsed into the software’s application programming interface (API) with supported diacritics from any language. A gender probability threshold of 60% was used to assign gender, consistent with previous studies [34,35]. Names with probabilities below this threshold (i.e., those for which prediction failed, or names deemed ambiguous by the software), were excluded from the analysis.

Our primary outcome was the proportion of studies with women in leadership, where leadership was defined as follows: ‘Woman first author only’ if the first author was a woman, and ‘Woman last author only’ if the last author was a woman. First and last authorship were analyzed as separate outcomes. If the first author’s name was missing, the entry was excluded from the first author analysis but retained for last author analysis when applicable (and vice versa). As a sensitivity analysis, the analyses for the primary outcome were repeated using gender probability thresholds of 70% and 80% for gender classification to assess the robustness of the findings to the choice of classification threshold. Secondary outcomes included the proportion of studies where both the first and last authors were women (‘Woman both first and last author’), and the proportion of studies where either the first or last author was a woman (‘Woman first or last author’), which combines the two primary outcomes into a single measure.

### Analysis

The characteristics of the included studies were analyzed using descriptive statistics, with categorical variables summarized as counts (percentages) and continuous variables summarized as means with standard deviations (SDs). We created two models for each analysis: one for first authors and one for last authors. We used Chi-square tests to assess differences in the proportion of women and men in leadership positions. We report differences in proportions as risk differences (RDs) with 95% confidence intervals (CIs). We estimated odds ratios (ORs) to test for associations between women’s leadership in methodological research and country income level, WHO region, number of authors on the study team, and publication year. For number of authors on the paper, we also reported the mean difference with the corresponding 95% CI. We created heat maps showing the percentage of women first and last authors by country, based on the location of their listed affiliation.

Given the potential for confounding among multiple publication factors, we built multivariable logistic regression models with woman first authorship and woman last authorship as binary outcomes, adjusting for country income level, WHO region, team size, and publication year. Adjusted odds ratios (aORs) with 95% CIs were estimated. Statistical significance was set at α = 0.05. All statistical analyses were performed using SAS, version 9.4 (SAS Institute Inc, Cary NC).

## Results

### Study characteristics

A total of 20,838 records were identified through the search. Following the removal of duplicates, 14,225 titles and abstracts were screened. From these, 2,123 full-text articles were assessed for eligibility, and 1,478 studies were initially included. However, some studies lacked a DOI and could not be used for name parsing, resulting in a final total of 1,375 eligible studies. The full flow of studies from the search is shown in S1 Fig.

To address missing data, 119 (8.7%) first author names and 320 (23.3%) first author countries were manually retrieved. Similarly, 133 (9.7%) last author names and 237 (17.2%) last author countries were important manually. In 37 studies (2.7%), the same individual was listed as both first and last author (i.e., single-author publications). Despite manual efforts, 6 first author names and 10 last author names could not be determined due to insufficient data from available sources. As a result, first author names were available for 1,369 studies and last author names for 1,365 studies. Author gender was determined for 1,357 first authors (12 unknown) and 1,354 last authors (11 unknown). Gender classification, based on a probability threshold of ≥60%, was successfully assigned to 1,332 (98.0%) first authors and 1,334 (98.4%) last authors, with publication years ranging from 1977 to 2020. The flow diagram illustrating the inclusion and exclusion of studies from the eligible 1,375 articles is shown in Fig 1.

**Fig 1:**
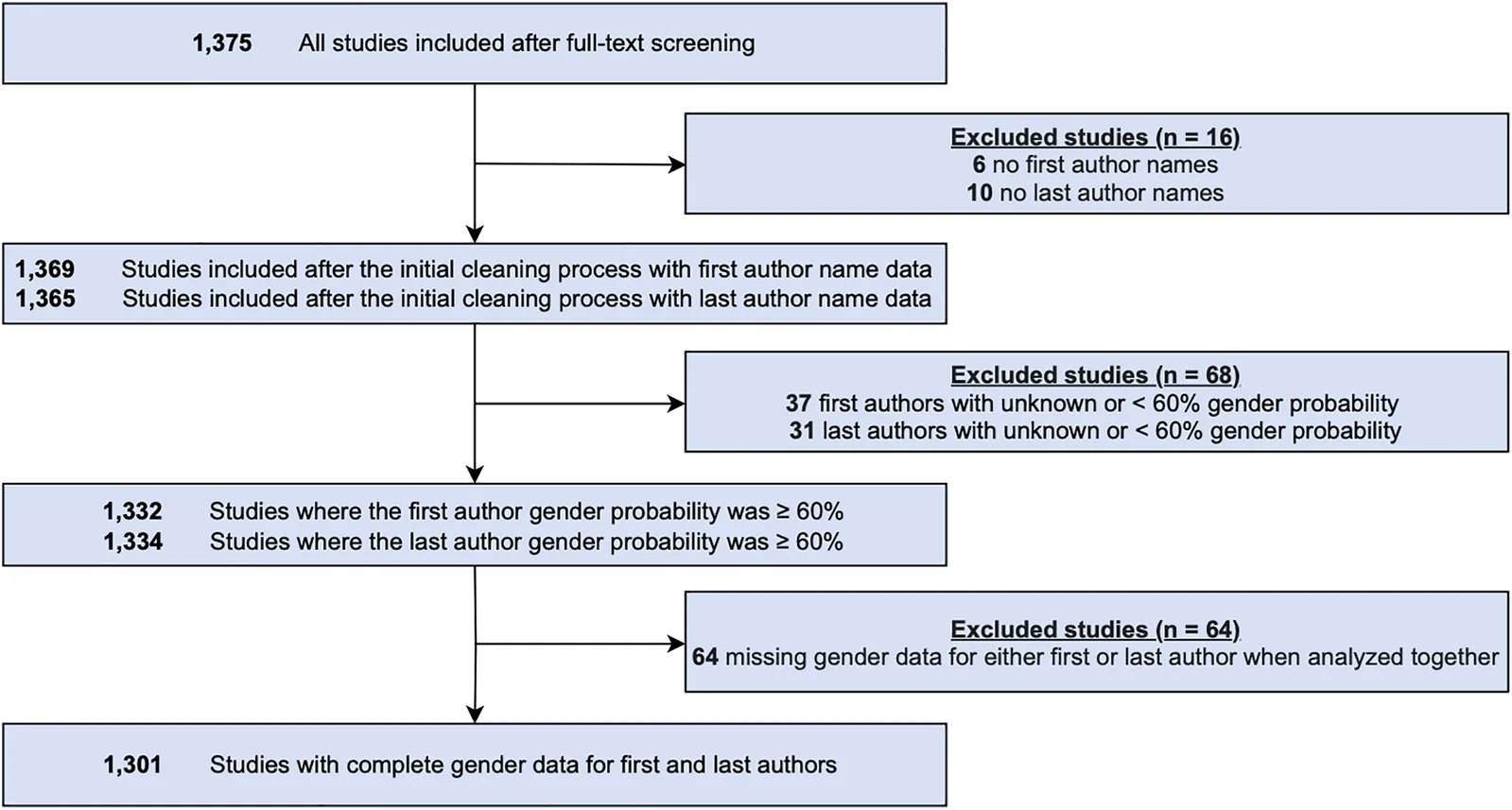
Flow diagram of methodological study selection for the analysis.

### Primary outcome

Among first authors, 675 (50.7%) were women, while 657 (49.3%) were men. For last authors, 486 (36.4%) were women, and 848 (63.6%) were men. The characteristics of included studies by first and last authorship are shown in Table 1. A Chi-square test revealed no statistically significant difference in the proportion of men and women as first authors (χ² = 0.24, p = 0.622; RD = 1.35%, 95% CI −2.44% to 5.15%). In contrast, a Chi-square test for last authors showed a significant gender difference (χ² = 98.23, p < 0.001; RD = −27.14%, 95% CI −32.50% to −21.77%).

**Table 1:** Characteristics of included studies by first and last author leadership.

First Author Leadership			
Characteristic	Woman (n = 675)	Man (n = 657)	Total (N = 1,332)
**Country Income Level: n (%)**			
Lower-middle income	8 (50)	8 (50)	16 (1.20)
Upper-middle income	45 (50.56)	44 (49.44)	89 (6.68)
High income	621 (50.69)	605 (49.39)	1,225 (92.04)
Missing	1 (100)	0 (0)	1 (0.08)
**WHO Region: n (%)**			
African Region	2 (25)	6 (75)	8 (0.60)
Region of the Americas	240 (52.29)	219 (47.71)	459 (34.46)
South-East Asia Region	3 (37.50)	5 (62.50)	8 (0.60)
European Region	345 (50.81)	334 (49.19)	679 (50.98)
Eastern Mediterranean Region	9 (56.25)	7 (43.75)	16 (1.20)
Western Pacific Region	75 (46.58)	86 (53.42)	161 (12.09)
Missing	1 (100)	0 (0)	1 (0.08)
**Study Team: mean (SD)**			
Number of Authors	5.86 (3.97)	5.67 (4.28)	5.76 (4.13)
**Last Author Leadership**			
**Characteristic**	**Woman** **(n = 486)**	**Man** **(n = 848)**	**Total** **(N = 1,334)**
**Income Level: n (%)**			
Lower-middle income	0 (0)	11 (100)	11 (0.82)
Upper-middle income	27 (36.99)	46 (63.01)	73 (5.47)
High income	459 (36.72)	791 (63.28)	1,250 (93.70)
**WHO Region: n (%)**			
African Region	2 (33.33)	4 (66.67)	6 (0.45)
Region of the Americas	197 (39.32)	304 (60.68)	501 (37.56)
South-East Asia Region	0 (0)	4 (100)	4 (0.30)
European Region	231 (34.58)	437 (65.42)	668 (50.07)
Eastern Mediterranean Region	2 (12.50)	14 (87.50)	16 (1.20)
Western Pacific Region	54 (38.85)	85 (61.15)	139 (10.42)
**Study Team: mean (SD)**			
Number of Authors	5.25 (3.28)	6.06 (4.53)	5.76 (4.13)

Abbreviations: standard deviation (SD); World Health Organization (WHO).

Sensitivity analyses using more stringent gender probability thresholds produced similar findings. There was no association at the 70% or 80% thresholds respectively (χ² = 0.02, p = 0.890; χ² = 0.03, p = 0.865). For last authors, at the 70% and 80% thresholds, the association remained statistically significant (χ² = 109.42, p < 0.001; χ² = 119.77, p < 0.001).

### Secondary outcomes

To assess the proportion of studies with women in leadership roles, we combined the data on first and last authors together and excluded studies with missing data. 1,301 studies had gender data for both first and last authors (Fig 1). Among these, 363 (27.9%) studies had a woman as the first author, 182 (14.0%) studies had a woman as the last author, 295 (22.7%) studies featured women as both first and last authors, and 545 (41.9%) studies had a woman as either first or last author. These results are illustrated in Fig 2.

**Fig 2:**
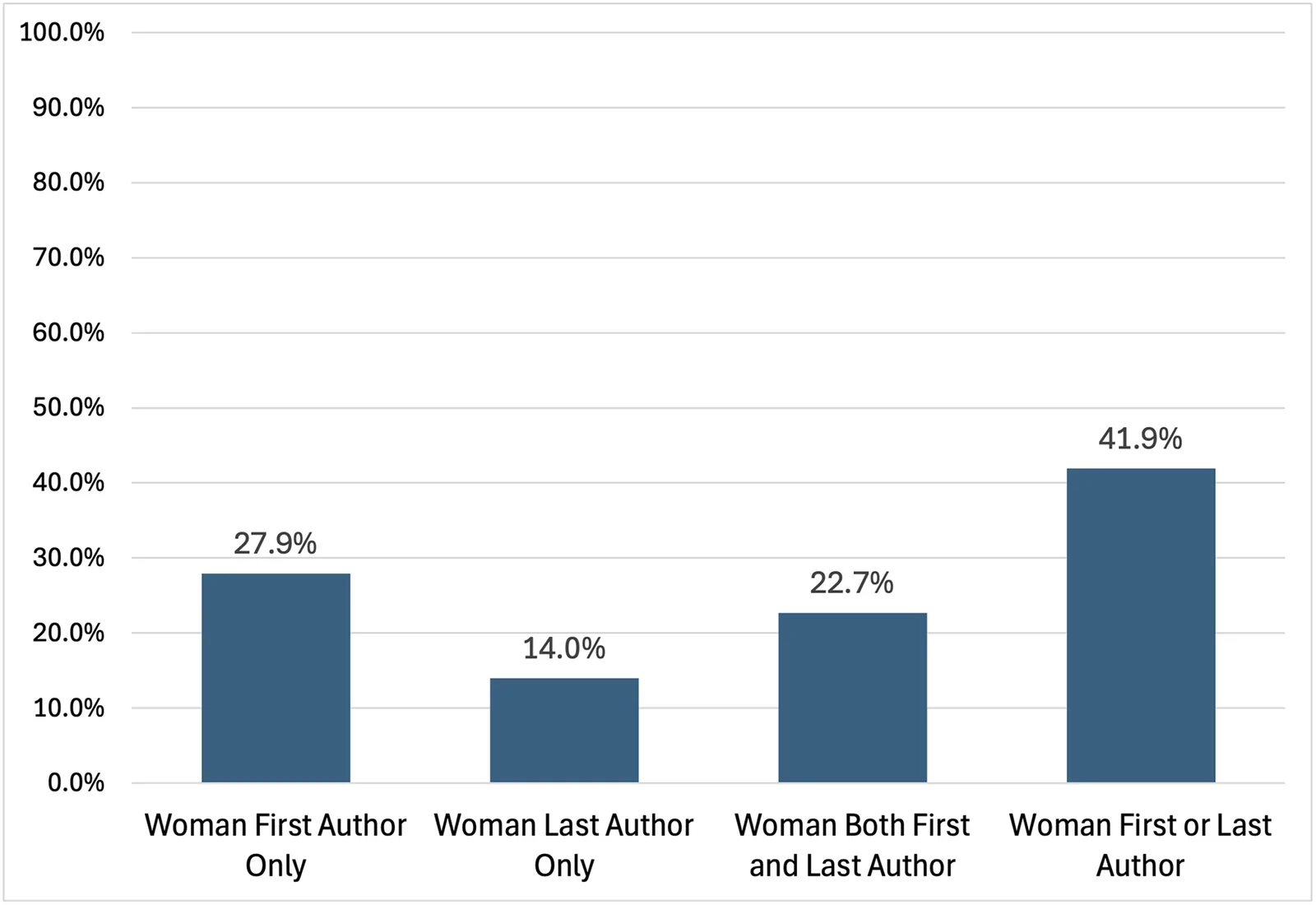
Percentage of studies with women’s leadership in methodological research.

### Factors associated with women’s leadership as first author

In unadjusted analyses, women’s leadership as first author was not associated with country income level or WHO region (Table 2). The mean (SD) number of authors per study in the first author dataset was 6 (4), with no statistically significant difference between studies with women as first authors and those with men as first authors (t = 0.82, p = 0.410). Publication year was associated with women first authorship, with each additional year associated with increased odds of women first authorship (OR=1.03, 95% CI 1.01–1.05).

**Table 2:** Unadjusted and adjusted logistic regression model results examining factors associated with woman first and last authorship.

First Author Woman				
Characteristic	OR (95% CI)^a^	p-value	aOR (95% CI)^a,b^	p-value
**Country Income Level**		**0.999**		**0.806**
Lower-middle income	0.97 (0.36-2.61)	0.959	1.37 (0.26-7.17)	0.712
Upper-middle income	1.00 (0.65-1.53)	0.988	1.17 (0.71-1.93)	0.545
High income	Reference	NA	Reference	NA
**WHO Region**		**0.522**		**0.355**
African Region	0.32 (0.07-1.61)	0.169	0.26 (0.05-1.39)	0.114
Region of the Americas	1.06 (0.84-1.35)	0.625	1.08 (0.85-1.38)	0.514
South-East Asia Region	0.58 (0.14-2.45)	0.459	0.43 (0.06-3.00)	0.396
European Region	Reference	NA	Reference	NA
Eastern Mediterranean Region	1.25 (0.46-3.38)	0.668	0.94 (0.24-3.72)	0.930
Western Pacific Region	0.84 (0.60-1.19)	0.335	0.78 (0.54-1.14)	0.202
**Number of Authors**	1.01 (0.99-1.04)	0.396	1.00 (0.97-1.03)	0.942
**Publication Year**	1.03 (1.01-1.05)	0.002	1.03 (1.01-1.05)	0.002
**Last Author Woman**				
**Characteristic**	**OR (95% CI)** ^**c**^	**p-value**	**aOR (95% CI)** ^**b,c**^	**p-value**
**Country Income Level**		**0.998**		**0.977**
Lower-middle income	–	0.972	–	0.982
Upper-middle income	1.01 (0.62-1.65)	0.963	0.94 (0.53-1.66)	0.831
High income	Reference	NA	Reference	NA
**WHO Region**		**0.260**		**0.475**
African Region	0.95 (0.17-5.20)	0.949	0.90 (0.15-5.46)	0.906
Region of the Americas	1.23 (0.96-1.56)	0.096	1.29 (1.01-1.65)	0.040
South-East Asia Region	–	0.974	–	0.988
European Region	Reference	NA	Reference	NA
Eastern Mediterranean Region	0.27 (0.06-1.20)	0.085	0.76 (0.14-3.97)	0.741
Western Pacific Region	1.20 (0.83-1.75)	0.339	1.15 (0.76-1.74)	0.510
**Number of Authors**	0.95 (0.92-0.98)	< 0.001	0.93 (0.90-0.97)	< 0.001
**Publication Year**	1.03 (1.01-1.05)	0.003	1.05 (1.02-1.07)	< 0.001

Abbreviations: adjusted odds ratio (aOR); confidence interval (CI); odds ratio (OR); World Health Organization (WHO).

^a^One observation with missing predictor data was excluded from the first author model (n = 1,331).

^b^Models are adjusted for country income level, World Health Organization (WHO) region, number of authors on the study team, and publication year.

^c^Estimates for the lower-middle income category and the South-East Asia Region are not shown because of complete separation (i.e., no woman last authors were observed in these categories).

In terms of geographic distribution, the three countries with the highest number of women first authors were the United Kingdom (150 studies, 22.3%), the United States (121 studies, 17.8%), and Canada (105 studies, 15.6%). The distribution of women’s leadership by country for first authors is shown in a heatmap (Fig 3).

**Fig 3:**
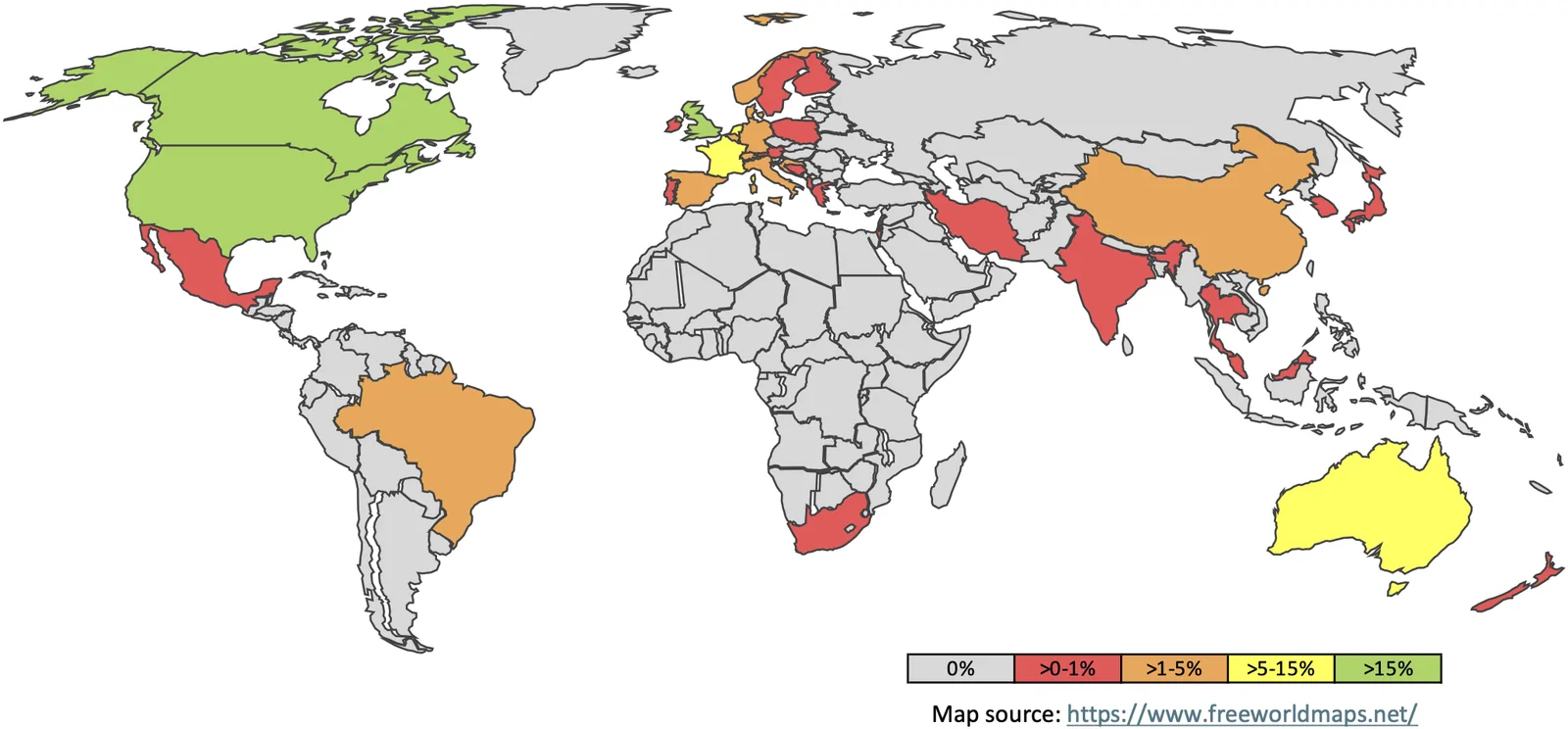
Heat map of the countries where women first authors are based, showing the percentage of studies in each country (n = 665).

In the adjusted analysis, country income level and WHO region were not significantly associated with the odds of women first authorship (Table 2). Number of authors on the study team was also not significantly associated with women first authorship (aOR=1.00, 95% CI 0.97–1.03). After adjustment, publication year remained significantly associated with women first authorship, with each additional year associated with increased odds of having a woman as first author (aOR=1.03, 95% CI 1.01–1.05).

### Factors associated with women’s leadership as last author

Women’s leadership as last author was not associated with country income level or WHO region. The mean (SD) number of authors per study in the last author dataset was 6 (4), with a statistically significant difference between studies with women as last authors and those with men as last authors (t = −3.77, p < 0.001), with a mean difference of −1 (95% CI: −1, −0.1). The odds of women last authorship increased with each additional year of publication (OR=1.03, 95% CI 1.01–1.05).

Similar to trends for first authorship, the three countries with the highest number of women last authors were the United Kingdom (111 studies, 22.8%), the United States (102 studies, 21.0%), and Canada (85 studies, 17.5%). The distribution of women’s leadership by country for last authors is shown in a heatmap (Fig 4).

**Fig 4:**
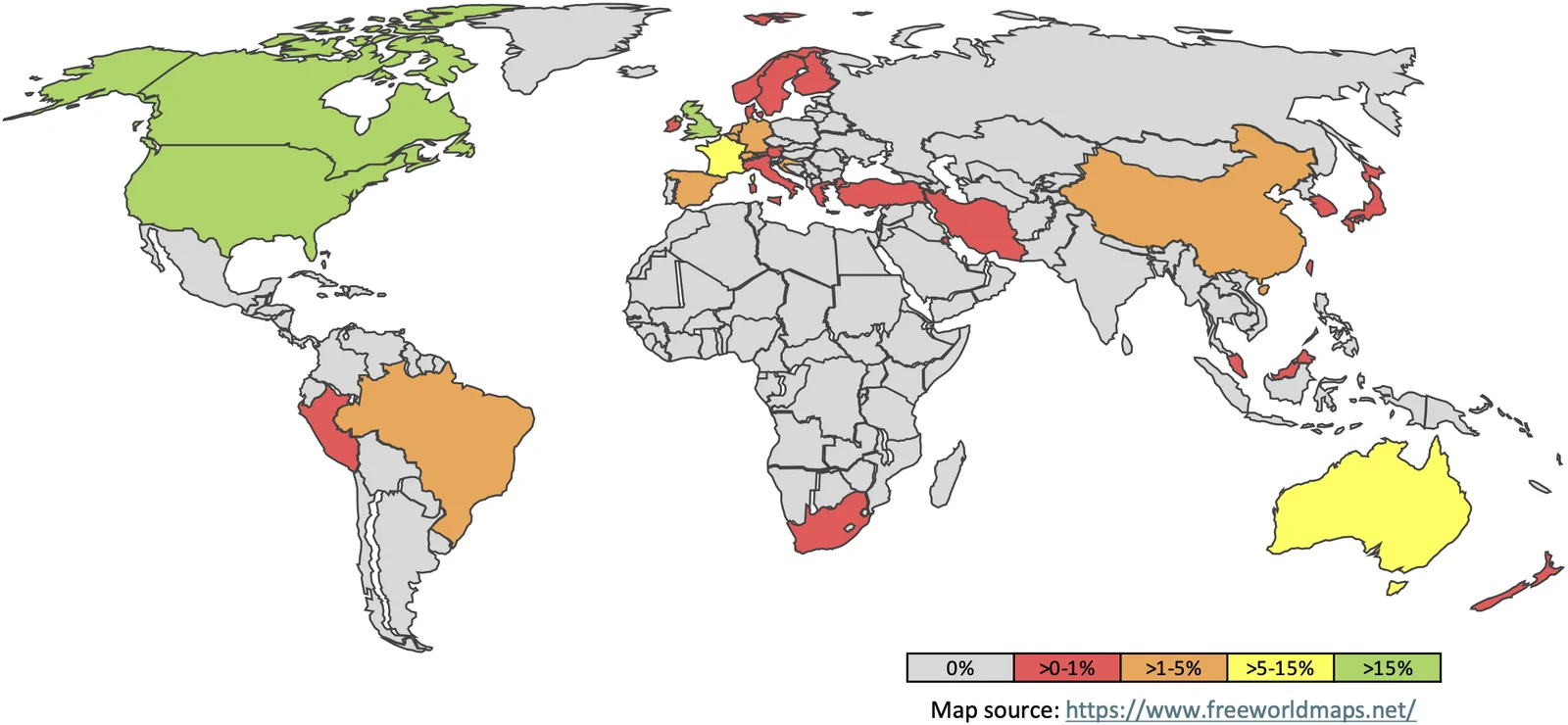
Heat map of the countries where women last authors are based, showing the percentage of studies in each country (n = 486).

In the adjusted analysis, country income level was not significantly associated with the odds of women being last author (Table 2). In contrast to the findings for first authorship, publications from the Region of the Americas had significantly higher odds of having a woman last author compared with those from the European Region (aOR=1.29, 95% CI 1.01–1.65), while no significant associations were observed for the other WHO regions. Number of authors on the study team was significantly associated with lower odds of women last authorship (aOR=0.93, 95% CI 0.90–0.97), while publication year was significantly associated with increased odds of women last authorship (aOR=1.05, 95% CI 1.02–1.07). Results from the unadjusted and multivariable logistic regression models for both first and last authorship are presented in Table 2.

## Discussion

This study provides valuable insights into gender disparities in leadership roles within methodological research in health science. To the best of our knowledge, it is the first study to assess gender distribution in first and last authorship roles within the field, key indicators of leadership in academic publishing. Our findings reveal a significant gender imbalance in the role of last authors (i.e., an indicator for seniority and mentorship), with men predominantly occupying these leadership positions. However, while gender inequality remains evident in leadership roles, our study also shows a more balanced gender distribution among first authors.

In adjusted models, factors associated with women’s representation differed by authorship position. WHO region was associated with women’s last authorship, with publications from the Region of the Americas showing higher odds of having a woman last author compared with those from the European Region, while no significant differences were observed across other regions. We also observed that for each added author on a publication, the odds of a woman occupying the last author position decreased. Publication year was significantly associated with both women first and last authorship, with each additional year associated with higher odds of women occupying these authorship positions. This result suggests that women’s representation in methodological research authorship has increased over time, although disparities in senior authorship remain.

Altogether, these findings align with previous literature where multiple studies have consistently shown that women are more frequently listed as first authors than last authors [36–39]. One study that examined gender biases in authorship in exercise and rehabilitation research found that women and men are nearly equally represented as first authors, however, gender bias against women persists in the last authorship position, irrespective of geographic region and journal ranking [40]. Another study highlights the variation in gender distribution among authors of global oncology publications across different regions and economic levels and found that female last authors were consistently underrepresented across every economic stratum, with the lowest representation observed in lower-middle income countries [41].

This pattern may reflect broader structural challenges faced by women researchers. A growing body of literature indicates that women researchers often encounter obstacles such as limited access to mentorship, networking, and funding opportunities, all of which hinder their ability to take on leadership roles [42,43]. In addition, the underrepresentation of women as last authors can be attributed to several factors. Gender disparity is particularly pronounced in the highest academic positions [20], where women receive fewer and smaller research grants than men [44,45], limiting their ability to lead higher impact research projects. Moreover, woman scientists often face various forms of discrimination that hinder their career progression, making it more difficult for them to attain senior academic roles and publish their research [36,46–49]. Our findings are likely driven by other factors that produce and reproduce these scientific gender disparities, including the reduced visibility and reputation of women academics. These factors include: women academics receiving fewer citations than men when listed as lead/senior authors in high-impact factor journals [15], reduced social media exposure [50], lower number of medical awards [51], lower number of leadership roles [52], reduced likelihood of being involved as content experts in media and press releases [53,54], lower involvement as speakers in grand rounds and international conferences [55,56], and bias in speaker introductions in Internal Medicine Ground Rounds where women are less likely to be introduced by professional titles [56]. The convergence of these systemic barriers continue to restrict their opportunities for leadership within the research community.

Our finding that women last authors were associated with smaller study teams compared to men last authors may reflect barriers that limit women’s access to larger, collaborative research efforts. Alternatively, they may prioritize including only those who make substantive contributions, while gender-based differences in collaboration practices could also influence team size. For example, some researchers may engage in broader collaborative networks, leading to larger author teams and potentially affecting publication patterns through reciprocal authorship invitations. Previous studies have suggested that the gender of the senior author may influence co-authorship patterns [57–59]. This is particularly important to address, as larger author teams may result in increased citations [60]. However, the relationship between gender and number of authors remains underexplored in the broader academic literature and warrants further investigation.

### Limitations

There are some limitations in our study that must be considered when interpreting our findings. These findings are based on predictive analytics, and we could only ascertain the expected gender of authors based on the algorithm Genderize.io uses. Future research should directly survey authors to improve the accuracy of gender-based analyses from published research. Furthermore, our study only categorized authors as either man or woman and did not account for non-binary genders. As a result, our analysis does not fully capture the diversity of gender identities within academic authorship. Also, the gender implied by a name does not necessarily reflect the gender that someone identifies as, further limiting our classifications. By limiting to a threshold of 60%, some authors were excluded from our analysis although less than 2.7% for first authors and 2.3% for last authors. To assess whether our findings were sensitive to the choice of threshold, we conducted a sensitivity analysis, which demonstrated that the findings for our primary outcome were robust to the selected gender probability threshold. Nevertheless, the use of probabilistic gender assignments remains subject to potential misclassification.

Gender-based stigma intersects with other socially marginalized categories (e.g., race, ability, sexual orientation, socio-economic status) and we were not able to ascertain the interaction between gender and these other categories when assessing authorship, thus we could not assess intersecting forms of bias [61]. Additionally, our analysis was reliant on publicly available information, and gender predictions could have been more accurate with additional demographic data, such as the age of authors. The inclusion of such demographic details would likely improve the accuracy of our gender prediction [33]. Further, we primarily focused on the gender distribution of the first and last authors, as these authorship positions are commonly used as proxies for key leadership roles in academic publishing, with the first author typically leading the study and the last author often representing the senior investigator. However, we acknowledge that authorship order is an imperfect measure of leadership, as conventions may vary across research teams and authorship order may not fully capture the complexity of leadership roles within methodological research teams. Accordingly, it remains unclear whether authorship order is a valid proxy for leadership in methodological research. Therefore, there may be value in examining the average proportions of female authors across studies to gain further insights on women’s contributions to methodological research. Lastly, differences in country affiliations between the first and last authors precluded our ability to analyze them together.

### Future work

Future research should investigate the multifaceted factors that contribute to the disparities observed in leadership within methodological research. Understanding these influences is crucial for addressing the barriers that limit women’s advancement in academic leadership, particularly in multi-author collaboration efforts. Additional points of interest for future work include examining gender distribution among middle authors, authors’ educational attainment, other socio-demographic factors, and journal impact factor.

## Conclusions

Our study reveals an important gender disparity in last author positions within methodological research in health science, with more men holding senior author roles than women. Additionally, we found that factors associated with women’s representation differed by authorship position. Women last authors were associated with smaller author teams, which may reflect differences in collaborative structures and access to leadership opportunities in larger-scale methodological research. Lastly, women’s representation in both first and last authorship positions increased over the study period, although women remained underrepresented in senior authorship roles. Addressing these challenges and advocating for research funding and policies that prioritize gender equity can help create a more inclusive research environment, especially within methodological research in health science.

## Supporting information

S1 Table Sample search strategy for MEDLINE. (DOCX)

S1 Fig Study flow diagram showing the screening and selection process of eligible articles. (PDF)
