# Supplementary material for: Gender disparities in leadership of methodological research in health science
Source: PLoS One. 2026 Sep 29;21(9):e0359403. doi: 10.1371/journal.pone.0359403 (PMC13623154; doi:10.1371/journal.pone.0359403)
Supplement: Supplementary file 1 [file pone.0359403.s002.pdf]

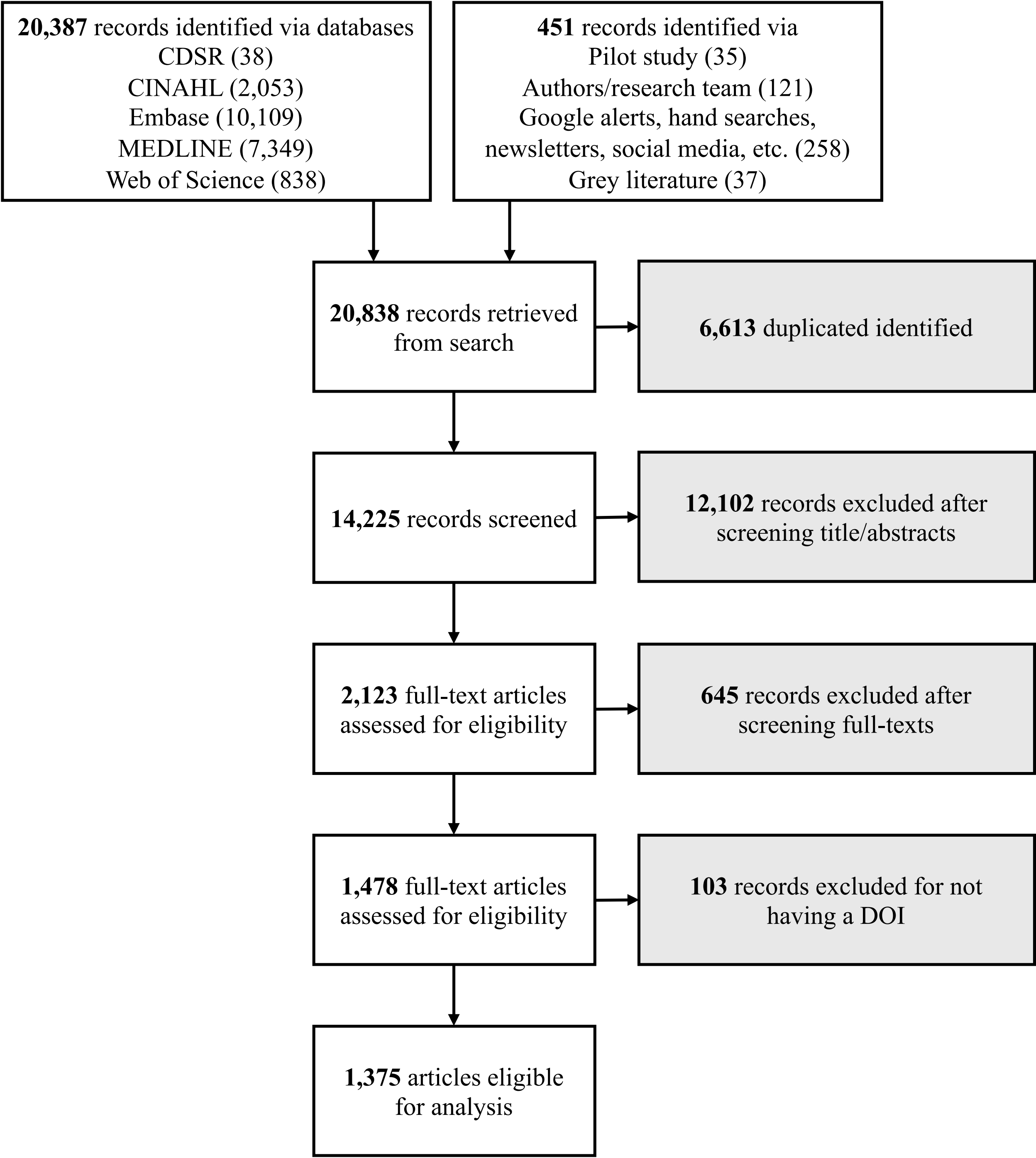
20,387 records identified via databases
CDSR (38)
CINAHL (2,053)
Embase (10,109)
MEDLINE (7,349)
Web of Science (838)
451 records identified via
Pilot study (35)
Authors/research team (121)
Google alerts, hand searches, newsletters, social media, etc. (258)
Grey literature (37)
20,838 records retrieved from search
6,613 duplicated identified
14,225 records screened
12,102 records excluded after screening title/abstracts
2,123 full-text articles assessed for eligibility
645 records excluded after screening full-texts
1,478 full-text articles assessed for eligibility
103 records excluded for not having a DOI
1,375 articles eligible for analysis
