# Supplementary material for: Gender disparities in leadership of methodological research in health science
Source: PLoS One. 2026 Sep 29;21(9):e0359403. doi: 10.1371/journal.pone.0359403 (PMC13623154; doi:10.1371/journal.pone.0359403)
Supplement: Supplementary file 2 [file pone.0359403.s001.docx]

**Table S1. Sample search strategy for MEDLINE.**

| Search Strategy | Search Terms |
| --- | --- |
| *Concept: names of methodological studies* | |
| 1 | (meta-epidemiolog* OR metaepidemiolog* OR meta-research OR methodolog* analysis OR methodolog* evidence OR methodolog* investigation OR methodolog* literature OR methodolog* overview OR methodolog* report* OR methodolog* review OR methodolog* survey OR methodolog* synthesis OR method* overview OR systematic database review OR systematic literature survey OR systematic survey).mp. |
| 2 | (methodolog* study OR method* review OR method* survey) |
| *Concept: topics in methodological research (i.e. analysis, design, and reporting)* | |
| 3 | exp Data Collection/ |
| 4 | exp Data Interpretation, Statistical/ |
| 5 | exp Epidemiologic Research Design/ |
| 6 | exp Nursing Methodology Research/ |
| 7 | exp Reproducibility of Results |
| 8 | exp Research Design/ |
| 9 | 3 OR 4 OR 5 OR 6 OR 7 OR 8 |
| 10 | 2 AND 9 |
| *Concept: methodological studies that are called ‘systematic reviews’* | |
| 11 | systematic review.mp. |
| 12 | Cochrane Database of Systematic Reviews.jn |
| 13 | 11 NOT 12 |
| 14 | *Data Collection/ |
| 15 | *Data Interpretation, Statistical/ |
| 16 | *Epidemiologic Research Design/ |
| 17 | *Nursing Methodology Research/ |
| 18 | *Reproducibility of Results/ |
| 19 | *Research Design/ |
| 20 | 14 OR 15 OR 16 OR 17 OR 18 OR 19 |
| 21 | 13 AND 20 |
| 22 | 1 OR 10 OR 21 |
